# Elucidating the Molecular Impact of DA29‐Induced ClpP Chemoactivation on Mitochondrial Function

**DOI:** 10.1096/fj.202603398RR

**Published:** 2026-10-17

**Authors:** Francesca Rizzo, Morena Miciaccia, Maria Grazia Perrone, Antonio Scilimati, Paola Loguercio Polosa, Francesco Bruni

**Affiliations:** ^1^ Department of Biosciences, Biotechnologies and Environment University of Bari ‘Aldo Moro’ Bari Italy; ^2^ Institute of Genetics and Molecular and Cellular Biology (IGBMC) Illkirch France; ^3^ Research Laboratory for Woman and Child Health, Department of Pharmacy—Pharmaceutical Sciences University of Bari ‘Aldo Moro’ Bari Italy

**Keywords:** ClpP, ClpX, DA29, DIPG, mitochondria, molecular therapy, ONC201, piperazine, protein quality control

## Abstract

Cellular function relies on tightly regulated mitochondrial proteolysis, which controls organelle protein quality and specific mitochondrial functions, including gene expression and oxidative phosphorylation. In human mitochondria, one of the main proteolytic machineries is the soluble ClpXP complex, consisting of the protease ClpP and its chaperone ClpX, which degrades misfolded or aggregated proteins to preserve organelle function and integrity. As hyperactivation of ClpP has been shown to inhibit tumor cell growth in various types of cancer, this protease has become an important target for the development of small‐molecule therapeutics. A piperazine‐based compound, DA29, has proven effective in inducing cytotoxicity in diffuse intrinsic pontine glioma (DIPG) patient‐derived cells and organoids, causing mitochondrial dysfunction and ROS accumulation. In this study, we employed *in cellulo* systems to conduct molecular and mechanistic analyses of ClpP hyperactivation mediated by DA29. Our data show that DA29 impairs mitochondrial function by specifically activating ClpP. Interestingly, degradation of ClpP itself is promoted when its proteolytic activity exceeds a certain threshold or is sustained for prolonged periods, suggesting a complex regulatory network that tunes protease levels within mitochondria.

## Graphical abstract

A promising approach to tackling DIPG, a lethal pediatric brain tumor, relies on small molecules capable of activating the human mitochondrial protease hClpP. The novel piperazine‐based compound DA29 specifically activates endogenous hClpP, thereby compromising mtDNA levels, mitoribosomes, and OXPHOS complex subunits. Our *in cellulo* system (HEK293T cells) reveals a long‐term response to DA29 mediated by a decrease of ClpP levels, consequently modulating the cellular response to protease activators.

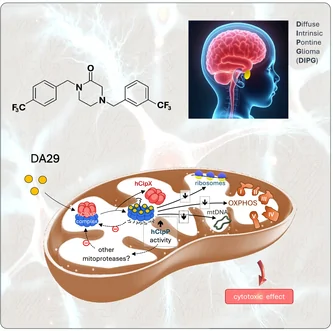

## 1 Introduction

Mitochondria, as essential cellular organelles, play a crucial role not only in cellular energy production through the oxidative phosphorylation (OXPHOS) system, but also in the regulation of different cellular processes such as apoptosis, calcium homeostasis, redox signaling, and stress response in either physiological or pathological conditions [1, 2].

Mitochondrial function depends on the nuclear DNA, as only 13 subunits of respiratory chain complexes are coded by the mitochondrial DNA (mtDNA). The remaining proteins, which in humans are estimated to be about 1200, are nucleus‐encoded, synthesized in the cytosol and then targeted to the submitochondrial compartments [3]. Recently, super‐resolution studies allowed a systematic mapping of the mitochondrial complexome from HEK293T cells, highlighting a multifunctional arrangement of mitochondrial protein machineries and interaction networks in human mitochondria [4]. Given this structural and functional complexity, nuclear and mitochondrial gene expression must be tightly coordinated to allow mitochondria to respond to variations in cellular energy demands and substrate availability.

Fine‐tuning of the mitochondrial function is important to maintain cellular homeostasis under stress conditions [2, 5]. This is provided by the mitochondrial unfolded protein response (UPRmt), which uses dedicated mitoproteases to degrade damaged proteins and activate rescue pathways such as mitophagy [6]. Beyond the quality control role, mitochondrial proteolysis directly regulates specific mitochondrial functions such as respiration, lipid biosynthesis, and dynamics. Moreover, mitoproteases can be modulated by a range of signaling elements, and their dysfunction can lead to various pathological conditions, such as cancer, metabolic syndromes, and neurodegenerative disorders [7].

A main component of the mitochondrial proteolytic system is the human caseinolytic protease P (ClpP). It is a soluble, highly conserved serine protease located in the mitochondrial matrix, consisting of a barrel‐shaped tetradecamer made by two stacked rings of seven subunits [8]. ClpP associates with the ATP‐dependent caseinolytic peptidase chaperone subunit X (ClpX) to form the ClpXP complex, which recognizes, unfolds, and threads misfolded proteins into the catalytic chamber [9]. Under normal physiological conditions, this tightly regulated proteolytic system ensures controlled protein turnover and adaptation to environmental stress. Additionally, ClpXP regulates several aspects of mitochondrial biogenesis such as (i) protein synthesis, (ii) synthesis of heme, (iii) OXPHOS activity and complex assembly, (iv) mtDNA maintenance and gene expression, possibly in concert with the matrix mitochondrial protease LON peptidase 1 (LONP1) [7, 10, 11].

Human ClpXP is upregulated in many primary and metastatic tumors and, in some cases, it correlates with shortened patient survival [12]. Because of its central role in proteostasis, ClpXP has emerged as a potential anticancer therapeutic target [13, 14]. ClpXP is directly bound by imipridone‐based compounds, such as ONC201, used for the treatment of patients affected by diffuse intrinsic pontine glioma (DIPG), which is a rare, aggressive pediatric brain tumor [15]. ONC201 binds to the hydrophobic pockets between ClpP subunits; this causes an allosteric change that widens the axial pore and promotes uncontrolled proteolysis [16]. By disrupting mitochondrial protein homeostasis, ClpP hyperactivation alters the organelle metabolism. Unfortunately, some DIPG patients have acquired resistance to this drug, raising the need to understand the molecular mechanisms underlying the development of this resistance and to design additional molecules [15].

Recent research has focused on the discovery and optimization of novel ClpP‐targeting small‐molecules with improved potency and cytotoxicity [17]. Among those molecules there are second‐generation imipridones, including the TR series, which exhibit higher ClpP activation potency than ONC201 [18]. Subsequent structural simplification of the TR scaffold led to the development of tetrahydropyridopyrimidindione derivatives, resulting in the THX and THY series, which display submicromolar activity in ONC201‐resistant cells [19]. More recently, further scaffold simplification generated the DA series, based on a piperazine core, with an improved pharmacokinetic profile [20]. Several studies, including ours, documented that the anticancer activity of these compounds could be linked to disruption of mitochondrial function [19, 20, 21]. However, a detailed mechanism explaining how these compounds kill cancer cells by hyperactivating proteolysis is still elusive.

In this study, to deepen the understanding of the cytotoxicity by ClpP chemoactivation, and to gain insights into proteostasis regulation in human mitochondria, we employed *in* *cellulo* systems such as HEK293T cells and the corresponding ClpP‐knockout (KO) line to perform biochemical and mechanistic analyses of ClpP hyperactivation by the piperazine‐based compound DA29.

## 2 Materials and Methods

### 2.1 Cell Cultures, Transfection and ClpP Expression

Wild‐type and ClpP‐knockout HEK293T (RRID:CVCL_0063) cells were cultured in Dulbecco's modified Eagle's medium (Thermo Fisher Scientific) supplemented with 10% (v/v) fetal bovine serum (FBS, Thermo Fisher Scientific), 1 mM sodium pyruvate, 1X non‐essential amino acids and 50 μg/mL uridine, in the presence of 50 U/mL penicillin and 50 μg/mL streptomycin at 37°C in humidified 5% CO_2_.

The plasmid constructs pcDNA5/FRT/TO/ClpP‐Hemagglutinin (HA) and pcDNA3.1/ClpP‐Green fluorescent protein (GFP), both including the N‐terminus signal peptide coding sequence, were commercially synthesized (GenScript). Transient transfections were carried out with Lipofectamine 3000 Transfection Reagent (Thermo Fisher Scientific), according to the manufacturer's instructions. The day following transfection, ClpP‐HA expression was induced by growing the cultures in the presence of 1 μg/mL tetracycline, whereas the fusion protein ClpP‐GFP was constitutively expressed. Cells were treated with ONC201 at a final concentration of 5.9 μM, DA29 and DA28 at a final concentration of 1.5 μM. Cellular confluency and ClpP‐GFP fluorescence were revealed by the EVOS M5000 Imaging System (Thermo Fisher Scientific).

At different times ranging from 2 to 6 days of treatment, whole‐cell extracts were prepared by solubilizing cell pellets in 50 mM Tris–HCl, pH 7.4, 150 mM NaCl, 1 mM EDTA, 1% Triton X‐100 and cOmplete Protease Inhibitor Cocktail (Roche), followed by incubation at 4°C for 30 min. Extracts were cleared by 15 min centrifugation at 12000 g at 4°C and stored at −20°C.

### 2.2 ClpP Activation Fluorescent Assay

For the ClpP proteolytic assay, mitochondrial lysates were prepared from both wild‐type and ClpP‐knockout HEK293T cells. After harvesting cells (400 g, 5 min, 4°C), pellets were resuspended in H‐buffer (0.6 M mannitol, 10 mM Tris–HCl, pH 7.4, 1 mM EGTA), freshly supplemented with 0.1% BSA (w/v) and subjected to standard differential centrifugation. Mitochondria were pelleted (11 000 g, 10 min, 4°C) and resuspended in ClpP lysis buffer (25 mM Tris–HCl, pH 7.4, 150 mM NaCl, 0.1 mM EDTA, 0.5% IGEPAL CA‐630 and 10% glycerol). The post‐mitochondrial supernatant was retained to analyze the cytosolic protein fraction for the subcellular localization experiment. After preparation, mitochondrial lysates were snap‐frozen and stored at −80°C.

ClpP protease activation was assessed by combining 500 fmol of fluorogenic protein substrate fluorescein‐isothiocyanate (FITC)‐casein (#C3777, Sigma‐Aldrich), 10 μg of mitochondrial lysate and the different compounds (ONC201, DA29 and DA28) at 100 μM final concentration. Reactions were set up in a 10‐μL final volume containing 25 mM HEPES‐KOH, pH 7.6, 5 mM MgCl_2_, 5 mM KCl, 0.03% Tween‐20, 10% glycerol and 2 mM DTT, and incubated at 37°C for 2 h. Samples were fractionated onto a 12% SDS‐PAGE minigel and fluorescent bands were detected by the Typhoon FLA 9500 phosphorimaging system (GE Healthcare Life Sciences). After detection, mitochondrial protein loading was determined by Coomassie blue staining.

### 2.3 Cytotoxicity Assay

The compounds DA28 and DA29 were dissolved in DMSO to prepare 10 mM stock solutions, from which a working solution was prepared in cell culture medium at a final concentration of 100 μM. Further dilutions were performed in cell medium to obtain final drug concentrations of 50, 25, 10, 1, and 0.1 μM. The assay was conducted by seeding cells at a density of 10 000 cells/well in a 96‐well plate (Corning) and culturing them for 24 h at 37°C in humidified 5% CO_2_. Subsequently, the culture medium was replaced with 100 μL of fresh medium containing the prepared dilutions of each compound; cells were incubated at 37°C up to 3 days. The highest DMSO concentration used in the assay (1% v/v) was tested separately as a vehicle control to evaluate potential solvent cytotoxicity. After incubation with each compound, CCK‐8 (10 μL) was added to each well, and after 3–4 h at 37°C, absorbance at *λ* = 450 nm was measured using the Infinite200 (Tecan) microplate reader. EC_50_ values were obtained by using GraphPad Prism software (RRID:SCR_002798).

### 2.4 Mitochondrial DNA Quantification

Total cellular DNA was isolated by standard phenol‐extraction and mtDNA relative levels were measured by real‐time qPCR using the SsoAdvanced Universal SYBR Green Supermix (Bio‐Rad) and the Applied Biosystems 7500 Fast Real‐Time PCR System (Thermo Fisher Scientific). Primer pairs were as follows: mtND4—forward: 5′‐CCATTCTCCTCCTATCCCTCAAC‐3′, reverse: 5′‐CACAATCTGATGTTTTGGTTAAACTATATTT‐3′; 18S rRNA gene—forward: 5′‐GTAACCCGTTGAACCCCATT‐3′, reverse: 5′‐CCATCCAATCGGTAGTAGCG‐3′; melting curve analysis was performed to ensure the amplification specificity. The relative quantification was carried out according to the formula *R* = (E_T_)^ΔCt,T^/(E_R_)^ΔCt,R^, where E_T_ = 1.94 and E_R_ = 1.87 indicated the amplification efficiencies on the mtND4 (target) and 18S rRNA gene (reference), respectively [22].

### 2.5 Isokinetic Sucrose Gradient

Whole‐cell lysates and mitochondrial extracts were prepared as described above and freshly complemented with 10 mM MgCl_2_ and 1 mM AEBSF. Aliquots (0.65–1 mg) were loaded onto a 10%–30% (v/v) sucrose gradient in 50 mM Tris–HCl, pH 7.2, 40 mM NH_4_Cl, 100 mM KCl, 10 mM Mg(OAc)_2_, 1 mM AEBSF and 50 μg/mL chloramphenicol. Gradients (2 ml) were prepared by Gradient Master (BioComp Instruments Ltd.) and subjected to centrifugation in an Optima L‐100 K ultracentrifuge (Beckman Coulter), using the SW 60Ti rotor at 32 000 rpm or at 40 000 rpm, for 2 h and 15 min at 4°C. For each protein sample, fractions (200 μL) were manually collected and stored at −20°C or, alternatively, at‐ −80°C (for longer preservation).

### 2.6 Immunoblotting Analysis

Whole‐cell proteins (20‐30 μg), mitochondrial lysates (30 μg) or sucrose gradient fractions (10 μL) were denatured in 4X Laemmli sample buffer (Bio‐Rad) complemented with 10% 2‐mercaptoethanol, separated by SDS‐PAGE and subjected to immunoblotting as described in [23], using the following primary antibodies: ClpP (#A306224, Antibodies.com), ClpX (#ab168338, Abcam), total OXPHOS cocktail (#ab110413, Abcam), HA‐tag (#3724, Cell Signaling Technology), uL13m (#16241–1‐AP, Proteintech), VDAC1 (#ab186321, Abcam), mS29 (#MA141279, Thermo Fisher Scientific), α‐tubulin (#T6074, Sigma‐Aldrich) and β‐actin (#A2066, Sigma‐Aldrich). Chemiluminescent signals were detected using the Clarity Western ECL substrate (Bio‐Rad) and the ChemiDoc MP Imaging System (Bio‐Rad). Densitometric analysis of band intensities was carried out by ImageJ 1.54p (RRID:SCR_003070) and GelQuant.NET 1.7.8 software.

### 2.7 L‐Lactate Assay

L‐lactate relative content in cell culture medium was assessed as described in [24], with modifications. Wild‐type and ClpP‐KO HEK293T cells were grown in 12‐well plates and treated with either DA29 or DA28 at a final concentration of 1.5 μM. After 3 days, cell media were collected and centrifuged at 10 000 g for 5 min. One μL of the supernatant was added to a 200‐μL reaction mixture containing 2.5 mM NAD^+^, 320 mM glycine, pH 9.5, 320 mM hydrazine and 0.6 U of bovine heart Lactate Dehydrogenase (LDH, Sigma‐Aldrich). Kinetics were carried out at 25°C and followed spectrophotometrically by Cytation 3 multi‐mode reader (BioTek) at 340 nm. For each sample, end‐point values were normalized to cell confluence.

## 3 Results

### 3.1 ClpP Mitochondrial Activity Was Enhanced by DA29

Several compounds, such as ONC201, have been shown to induce mitochondrial dysfunction and cytotoxicity by hyperactivating the ClpP protease [21]. To evaluate the direct effect of activator compounds on mitochondrial ClpP activity, a gel‐based protease assay was set up using FITC‐casein as the substrate, and a mitochondrial lysate as the primary source of ClpP proteolytic activity. Firstly, we prepared mitochondrial lysates from wild‐type and ClpP‐KO HEK293T cells and confirmed that ClpP was absent in the KO lysates (Figure 1A). Since mitochondria were extracted and lysed under mild conditions, the ClpXP complex most likely retained its assembled state within the lysate. To confirm this, the mitochondrial lysate from wild‐type cells was fractionated on an isokinetic gradient, and the distribution of ClpP and ClpX across the fractions was assessed. As shown in Figure 1B, ClpP and ClpX co‐sedimented in the same fractions (lanes 1–4), also forming high‐molecular‐weight complexes comparable to those of the small and large ribosomal subunits (fractions 5–7), represented by mS29 and uL13m, respectively. The observed pattern was suggestive of an association between ClpP and its chaperone within the same complexes.

**FIGURE 1:**
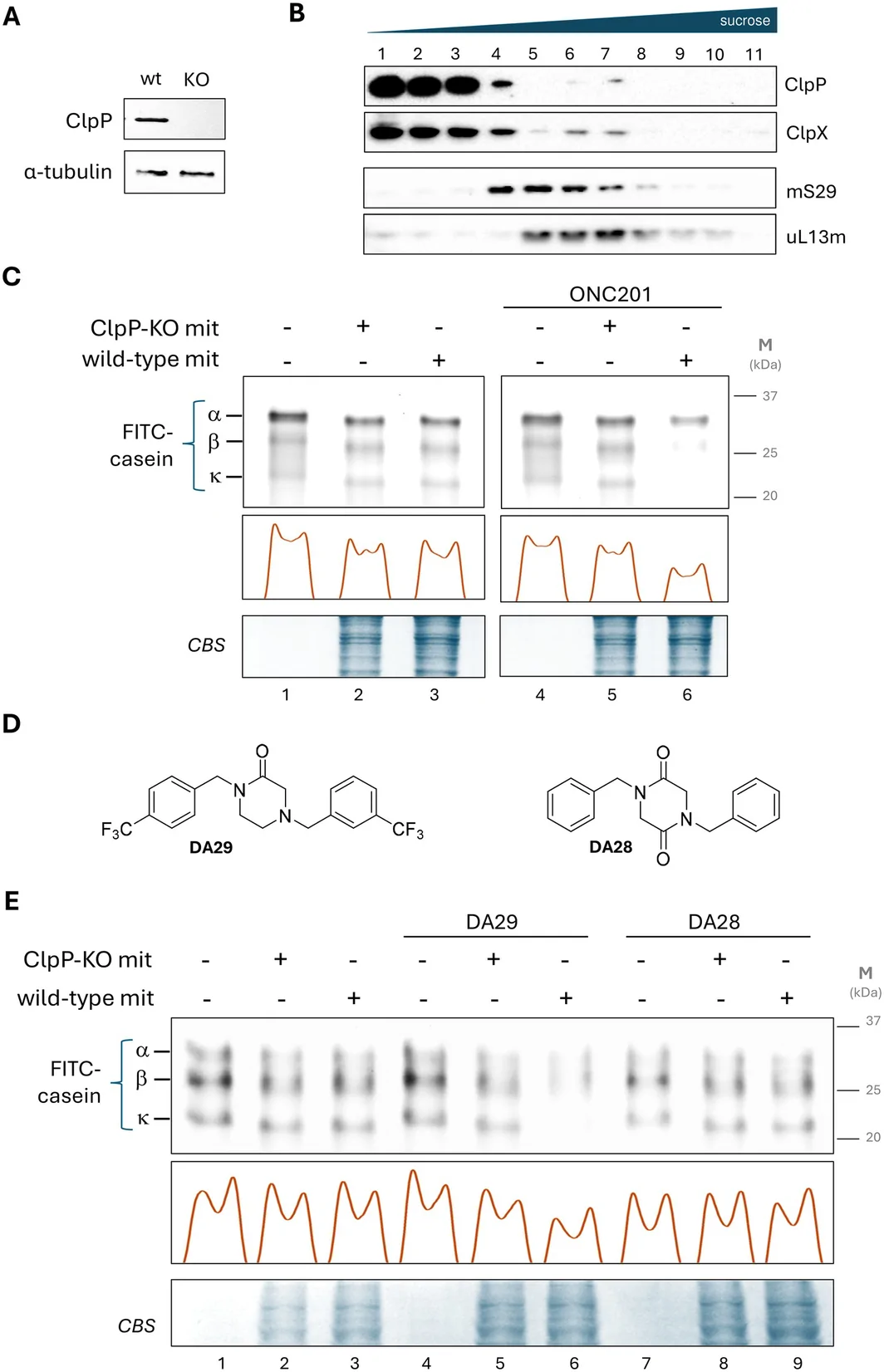
Targeted activation of endogenous mitochondrial ClpP. (A) Western blots of mitochondrial lysates (30 μg) from wild‐type and ClpP‐KO HEK293T cells were probed with antibodies against ClpP and α‐tubulin, the latter being used as a loading control. (B) Analysis of ClpP and ClpX assembly was carried out in wild‐type HEK293T mitochondria through isokinetic sucrose gradient centrifugation. Mitochondrial lysate (0.65 mg) was centrifuged on a 10%–30% gradient using the SW 60Ti rotor at 40 000 rpm for 2 h and 15 min at 4°C. Small and large mitoribosomal subunits were used as a reference of migration and were immunodecorated by antibodies against marker proteins of the mt‐SSU (mS29; HUGO protein name: DAP3) and the mt‐LSU (uL13m; HUGO protein name: MRPL13). (C) Effect of ONC201‐mediated ClpP activation on casein hydrolysis in the PAGE‐based fluorescent assay. Mitochondrial lysates (50 μg) from wild‐type and KO cells were used as a source of ClpP activity, and incubated with ONC201 at a final concentration of 100 μM. Upper panels: Milk casein isoforms (α, β and k) are depicted to the left; molecular weight markers (M; Precision Plus Protein Dual Color Standards, Bio‐Rad) are indicated to the right. Middle panels: Total densitometric profile of fluorescent substrate was determined for each lane. Lower panels: Coomassie blue staining (CBS) of mitochondrial proteins was used as a loading control. (D) Chemical structure of DA29 (ClpP activator) and DA28 (negative control). (E) Effect of ClpP chemoactivation by DA29 on casein hydrolysis in the PAGE‐based fluorescent assay; DA28 was used as a negative control. For description, see panel C.

The FITC‐casein assay was refined in the absence and presence of ONC201, a well‐characterized potent ClpP activator that has been recently approved by the Food and Drug Administration (FDA) for a systemic therapy for H3 K27M‐mutant diffuse midline glioma [25]. In the absence of ONC201, the endogenous mitochondrial ClpP protease activity was extremely low, with no substantial degradation of FITC‐casein compared to the control (Figure 1C, lane 3 vs. lane 1). The addition of ONC201 activated ClpP, resulting in effective degradation of the fluorescent substrate (Figure 1C, lane 6 vs. lane 4). To verify that casein degradation was exclusively mediated by ClpP and not by other mitochondrial proteases, the substrate was incubated with a mitochondrial lysate obtained from ClpP‐KO HEK293T cells. As shown in Figure 1C (lanes 2 and 5), ClpP‐KO cells were not proteolytically active, even in the presence of ONC201; hence, the assay was specifically sensitive to the activation of ClpP.

Structural and mechanistic studies have shown that DA29, a piperazine‐2‐one (endocyclic carbonyl)‐based ClpP activator (Figure 1D, left), binds to the hydrophobic pocket of ClpP, thereby inducing mitochondrial dysfunction and cytotoxicity in patient‐derived DIPG cells and organoids [20]. Here, the ability of DA29 to stimulate the proteolytic activity of ClpP in its physiological context was assessed by FITC‐casein protease assay. Another piperazine‐based compound, named DA28 (Figure 1D, right), which was unable to bind ClpP and induce its activity [20], was used as a negative control. We found that DA29, similarly to ONC201, was able to activate ClpP and degrade the casein (Figure 1E, lane 6); as expected, the mitochondrial activity of the protease did not change in the presence of DA28 (lane 9), and mitochondrial lysates from ClpP‐KO cells (lanes 2 and 5) did not show proteolytic activity, even in the presence of DA29. Overall, our data indicate that the basal activity of ClpP from mitochondrial lysates was not sufficient to degrade casein; importantly, ONC201 and DA29 were able to specifically activate the proteolysis mediated by endogenous ClpP.

### 3.2 DA29 Negatively Affected HEK293T Cell Growth

Data obtained on patient‐derived DIPG cells demonstrate that ClpP activators exhibit high toxicity even at sub‐micromolar concentrations [15]. While this is an advantage for treatment from a pharmacokinetic standpoint, it nevertheless represents a limitation for the in‐depth molecular characterization of the cytotoxicity mechanisms using cultured DIPG cells. For this reason, we explored the impact of these compounds on cell growth using human HEK293T cells, which constitute a well‐known cellular model and allow the analysis of different phenotypes through depletion and overexpression of the ClpP protease.

Wild‐type and ClpP‐KO HEK293T cells were treated with DA29, ONC201 (positive control) and DA28 (negative control) for up to 3 days, and the cell confluency was monitored (Figure 2A). DA29, as well as ONC201, significantly impaired the growth of wild‐type cells as early as 24 h, while DA28 did not alter the growth rate of these cells compared to untreated cells (Figure 2A, left panel); as expected, in ClpP‐KO cells neither of the compounds had any effect (Figure 2A, right panel).

**FIGURE 2:**
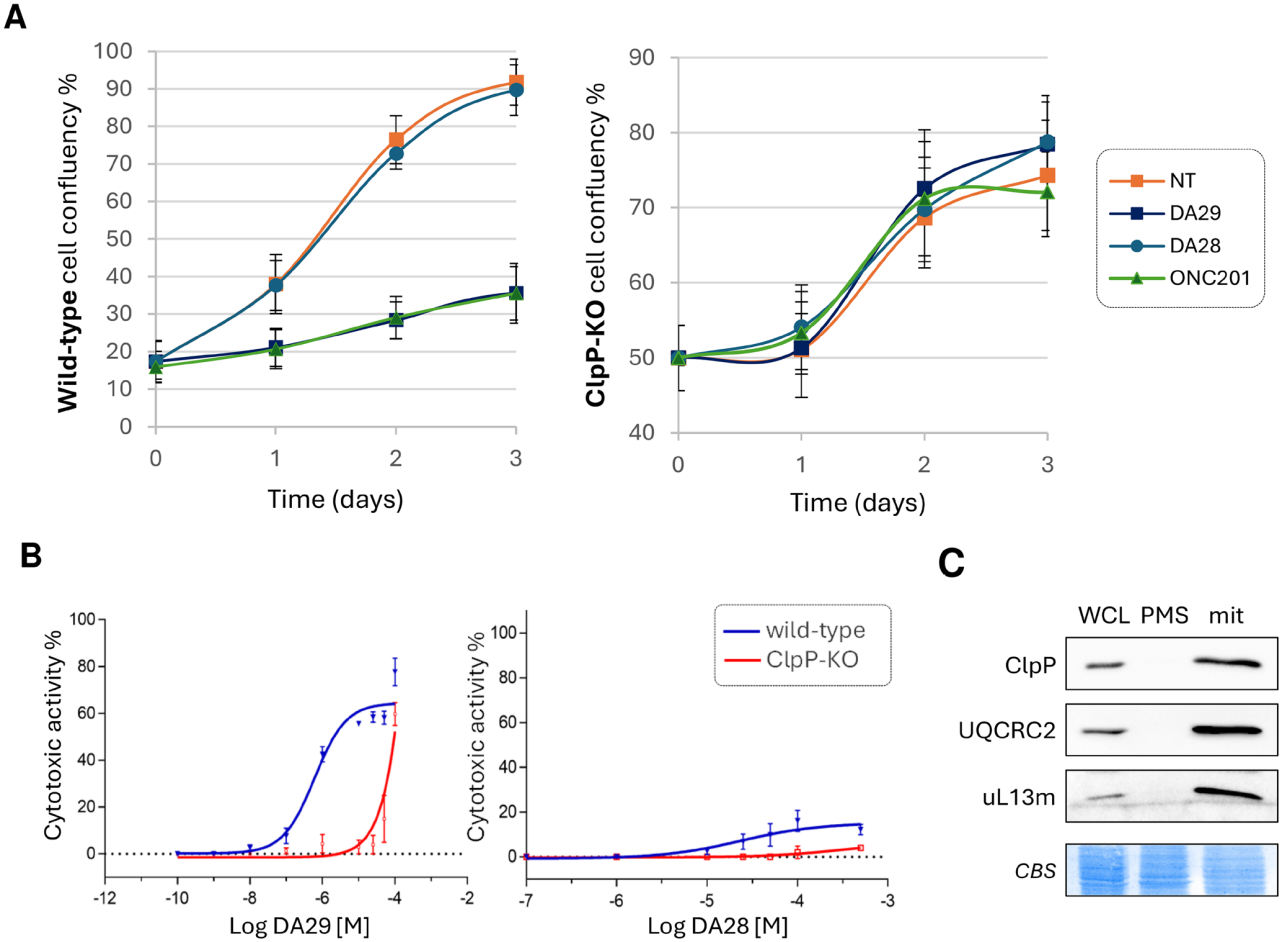
Cell viability upon DA29 treatment and subcellular ClpP localization. (A) Time‐courses of wild‐type (left panel) and ClpP‐KO (right panel) cell growth. Cells were either untreated (NT) or treated with ONC201 (5.9 μM), DA29 (1.5 μM) or DA28 (1.5 μM). Wild‐type and KO cells were seeded at different initial confluencies of 20% and 50%, respectively. This difference reflects their distinct growth rates, with ClpP‐depleted cells growing more slowly. Results are presented for each time point as the mean ± standard deviation (SD) of triplicates from three independent experiments (*n* = 9). (B) Cytotoxic dose–response curves of wild‐type and ClpP‐KO cells treated with compounds DA29 (left) and DA28 (right), according to dilution scheme detailed in Section 2.3. Data were obtained by CCK‐8 assay; cell viability in untreated cells ([compound] = 0) was set to 100%, and cytotoxic activity (%) was calculated as [100 − cell viability (%)]. As a vehicle control, the highest DMSO concentration used in the assay (1% v/v) was tested separately and showed comparable cell viability to untreated cells. Data are expressed as the mean ± SD of three independent experiments. (C) Mitochondrial localization of human ClpP. Aliquots (30 μg) of HEK293T whole‐cell lysate (WCL), post‐mitochondrial fraction (PMS) and mitochondria (mit) were subjected to immunoblotting with specific antibodies against ClpP and two mitochondrial marker proteins, UQCRC2 (inner mitochondrial membrane) and uL13m (mitochondrial matrix). CBS of the membrane was used as a loading and transfer control.

The cytotoxicity of DA29 and DA28 was also evaluated on both wild‐type and ClpP‐KO HEK293T lines after 3 days of treatment (Figure 2B). DA29 was much more effective in the wild‐type cell line (EC_50_ = 0.6 ± 0.27 μM) compared to knockout cells (EC_50_ > 100 μM). The latter, however, showed a slight rise in cytotoxicity at the highest DA29 concentration (Figure 2B, left panel), possibly due to secondary off‐target biological effects in the presence of very high doses of DA29 compound. Treatment with DA28 did not cause appreciable cytotoxicity in both cell lines (Figure 2B, right panel). The above results showed that cells lacking ClpP were insensitive to the effect of DA29 at the treatment concentration, confirming that ClpP is a specific cellular target of this activator.

Evidence from various experimental approaches indicates that ClpP is primarily localized within mitochondria [26, 27, 28] and forms a soluble protease complex with ClpX in the mitochondrial matrix. To rule out potential extra‐mitochondrial effects resulting from DA29 targeting active ClpP precursors in the cytosol, cells were fractionated and analyzed by immunoblotting (Figure 2C). ClpP co‐localized with the mitochondrial proteins UQCRC2 (core subunit of respiratory Complex III) and uL13m (component of the mitochondrial large ribosomal subunit) but was absent in the cytosolic fraction, confirming its mitochondrial localization.

### 3.3 ClpP Specifically Mediated the DA29 Effect on Mitochondrial Function

The ability of DA29 to activate endogenous ClpP in HEK293T mitochondria prompted us to evaluate its effect on mitochondrial processes.

First, we determined the effect of ClpP chemoactivation by DA29 on mitochondrial (mt) DNA relative levels in HEK293T wt and ClpP‐KO cells. Consistently with data from patient‐derived DIPG cells [20], DA29 caused a strong reduction of mtDNA in wild‐type cells, unlike DA28 (Figure 3A, left panel). In ClpP‐KO cells, both compounds induced a non‐statistically significant decrease in mtDNA relative levels, possibly ascribed to ClpP‐independent effects (Figure 3A, right panel).

**FIGURE 3:**
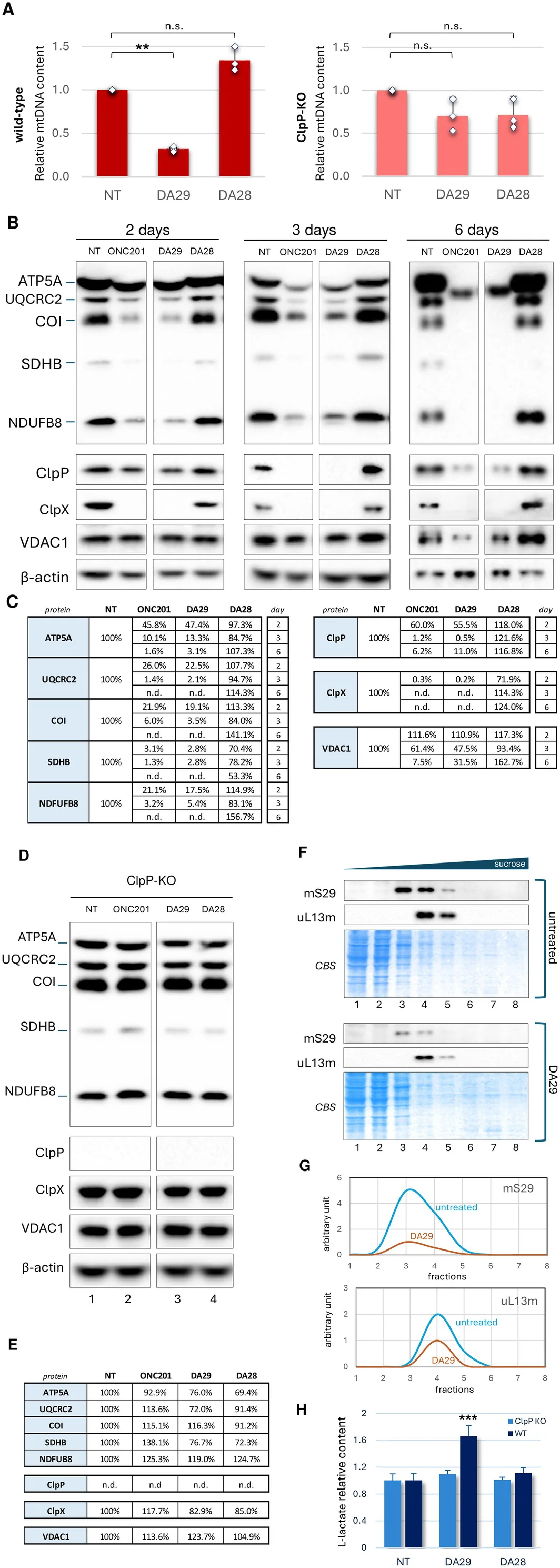
ClpP‐mediated DA29 effect on mitochondrial function. (A) Relative mtDNA content of wild‐type (left panel, dark red bars) and ClpP‐KO (right panel, light red bars) cell lines, both treated with either DA29 or DA28, compared to untreated (NT) cells. Data are expressed as the mean ± S.D. and statistical analyses were performed on three independent replicates using two‐tailed Student's *t*‐test. Asterisks (**, *p* < 0.001) indicate the significance of variations in treated samples (DA29 and DA28) with respect to the untreated (NT); n.s., not significant. Individual data points are depicted by white diamond‐shaped dots. (B) Whole‐cell lysates were prepared from untreated (NT) and compound‐treated wild‐type cells at various times (2, 3 and 6 days). Aliquots (20 μg) were subjected to immunoblotting, as detailed in Section 2.6. Data were obtained from three independent experiments, one for each time point. (C) Relative quantification of proteins shown in panel B. For each protein at the various time points, densitometric analysis was performed on the bands corresponding to the different treatments (ONC201, DA29, and DA28), normalized to β‐actin (loading control) and compared to the untreated (NT) sample (fixed as 100%); n.d., indicates not detectable signal. (D) Whole‐cell lysates were prepared from ClpP‐KO cells untreated (NT) and treated with different compounds for 3 days. Aliquots (20 μg) were subjected to immunoblotting, as detailed in Section 2.6. (E) Relative quantification of proteins shown in panel D. For each protein, densitometric analysis was carried out as described in (C). (F) Isokinetic sucrose gradient followed by immunoblot analysis of mitoribosomal proteins mS29 and uL13m in wild‐type cells either untreated (upper panel) or treated with DA29 (lower panel), as depicted to the right. Whole‐cell lysate (1 mg) was centrifuged on a 10%–30% gradient using SW 60Ti rotor at 32 000 rpm for 2 h and 15 min at 4°C. CBS of membranes was used as a loading and transfer control. (G) The abundance profile of mS29 and uL13m was determined by densitometric analysis of immunoblots shown in panel F, relative to untreated (blue line) and DA29‐treated (red line) cells, normalized to protein content of each fraction. The data are expressed as arbitrary units (treated/untreated ratio). (H) Extracellular lactate relative levels were measured in growth media from either wild‐type (dark blue bars) or KO (light blue bars) cells, normalized to cellular confluency and expressed as fold change between cells treated for 3 days and untreated (NT) control, the latter fixed as 1‐value. Results are presented as the mean ± SD of triplicates from three independent experiments (*n* = 9). Statistical analysis was performed using the two‐tailed Student's t tests. Asterisks (***, *p* < 0.0001) indicate the significance of variations in DA29‐treated cells in relation to the respective untreated (NT). Final concentrations of the compounds for all the experiments shown in Figure 3 were as follows: ONC201 (5.9 μM), DA29 (1.5 μM) and DA28 (1.5 μM).

The observed decrease in the relative mtDNA copy number in the presence of DA29, and the already known regulatory role of ClpP on the levels of several mitochondrial proteins, prompted us to measure the effect of DA29 on the steady‐state level of some respiratory chain subunits such as NDUFB8 (NADH dehydrogenase [ubiquinone] 1 beta subcomplex subunit 8; Complex I), SDHB (Succinate dehydrogenase [ubiquinone] iron–sulfur subunit; Complex II), UQCRC2 (Ubiquinol‐cytochrome c reductase core protein 2; Complex III) and ATP5A (ATP synthase F(1) complex subunit alpha; Complex V), which are all nuclear‐encoded subunits, and COI (Cytochrome c oxidase subunit 1; Complex IV), which is a mtDNA‐encoded subunit. After 2, 3, and 6 days of treatment with DA29, we found a marked progressive decline of all subunits' levels (Figure 3B,C). A similar general pattern of decrease was observed following treatment with ONC201, whereas DA28 had no substantial effect. We also assessed the integrity of the ClpXP complex. It is known, in fact, that following the binding of activators to the ClpP hydrophobic pockets, the hexameric ring of ClpX is displaced from the protease [29]; a concomitant loss of ClpX occurs [19, 20, 30, 31] through a mechanism that has not yet been clarified. After 2 days of treatment with either ONC201 or DA29, we observed the loss of ClpX as well as a 40%–45% decrease of ClpP levels (Figure 3B,C). Importantly, after 3 days ClpP was undetectable in cells treated with either ONC201 or DA29, becoming barely detectable again after 6 days of administration; DA28 treatment, however, had no effect. Furthermore, after 3 and 6 days we observed a progressive decrease of VDAC1 (Voltage‐dependent anion‐selective channel protein 1), a marker of mitochondrial mass; this reduction, consistently with the concomitant mtDNA depletion, may point to a decline in mitochondrial biogenesis. In contrast, administration of ONC201 and DA29 to ClpP‐KO cells did not perturb either respiratory chain complexes or steady‐state levels of ClpX and VDAC1 (Figure 3D,E).

Mitoribosomes serve as a critical hub for mitochondrial protein quality control [32]. Given that ClpP mediates the degradation of many mitochondrial ribosomal proteins (MRPs) and regulates mitoribosomal assembly [33], we evaluated the status of ribosomal subunits in both untreated and DA29‐treated cells. Analysis of their distribution across isokinetic gradients (Figure 3F), based on immunodetection of both mS29, representative of mitochondrial small ribosomal subunit (mt‐SSU; fractions 3–4), and uL13m, representative of mitochondrial large ribosomal subunit (mt‐LSU; fractions 4–5), showed an overlapping distribution profile in untreated and DA29‐treated cells (Figure 3G). The observed pattern suggests that no alteration of ribosomal subunits' assembly occurred following ClpP chemoactivation. The fainter signals in DA29‐treated cells compared to untreated indicate that the steady‐state levels of both representative MRPs, uL13m and mS29, were decreased (Figure 3F,G). These findings confirm that ClpP hyperactivation likely results in a reduction of the ribosome pool available for mitochondrial protein synthesis, consistent with observations previously reported in patient‐derived DIPG cells [20].

Finally, it is widely recognized that cells switch to anaerobic glycolysis when they cannot use aerobic metabolism to efficiently produce ATP, resulting in the accumulation of lactate that links glycolysis and mitochondrial respiration. Considering the here reported negative effect of ClpP hyperactivation on the respiratory chain, we measured the relative amount of lactate in both wild‐type and ClpP‐KO cells, untreated and treated with DA29. Analysis of extracellular medium showed a significant lactate increase in wild‐type cells treated with DA29, but not with DA28, with respect to untreated (Figure 3H). In contrast, no lactate accumulation occurred in KO cells, confirming that the glycolytic switch, paralleled by mitochondrial dysfunction, was due to ClpP hyperactivation by DA29.

### 3.4 Exogenous Expression of ClpP in Knockout Cells Mimicked the Effect of Activators

The observation that ClpP activation promoted its own degradation (see Figure 3B) led us to further investigate the impact of modulating the protease levels on mitochondrial function. Our experimental model consisted of ClpP‐KO cells overexpressing an HA‐tagged version of ClpP, which were subsequently exposed to DA29. To this aim, ClpP‐KO cells were transiently transfected with a ClpP‐HA construct including the natural mitochondrial targeting sequence; both untransfected and transfected cells were then treated with DA29 for 2 days. Notably, ClpP overexpression is not conceptually equivalent to ClpP activation and is not free from non‐physiological effects. Despite these limitations, this system has been successfully used to demonstrate the suppressive effects of ClpP overexpression on tumor growth in vivo, which paralleled the aberrant ClpP chemoactivation [34]. In our model, we observed that ClpP overexpression in untreated cells caused a general decline in respiratory subunits and VDAC1 levels (Figure 4A, lane 3 and Figure 4B), similarly to what observed in DA29‐treated wild‐type cells (see Figure 3B,C); ATP5A and SDHB were not substantially affected. Overexpressed ClpP, hyperactivated by DA29, induced its own degradation as also observed in wild‐type cells treated for 3 and 6 days (see Figure 3B,C) but also promoted a partial rescue of respiratory subunits and VDAC1 (Figure 4A, lane 4 and Figure 4B).

**FIGURE 4:**
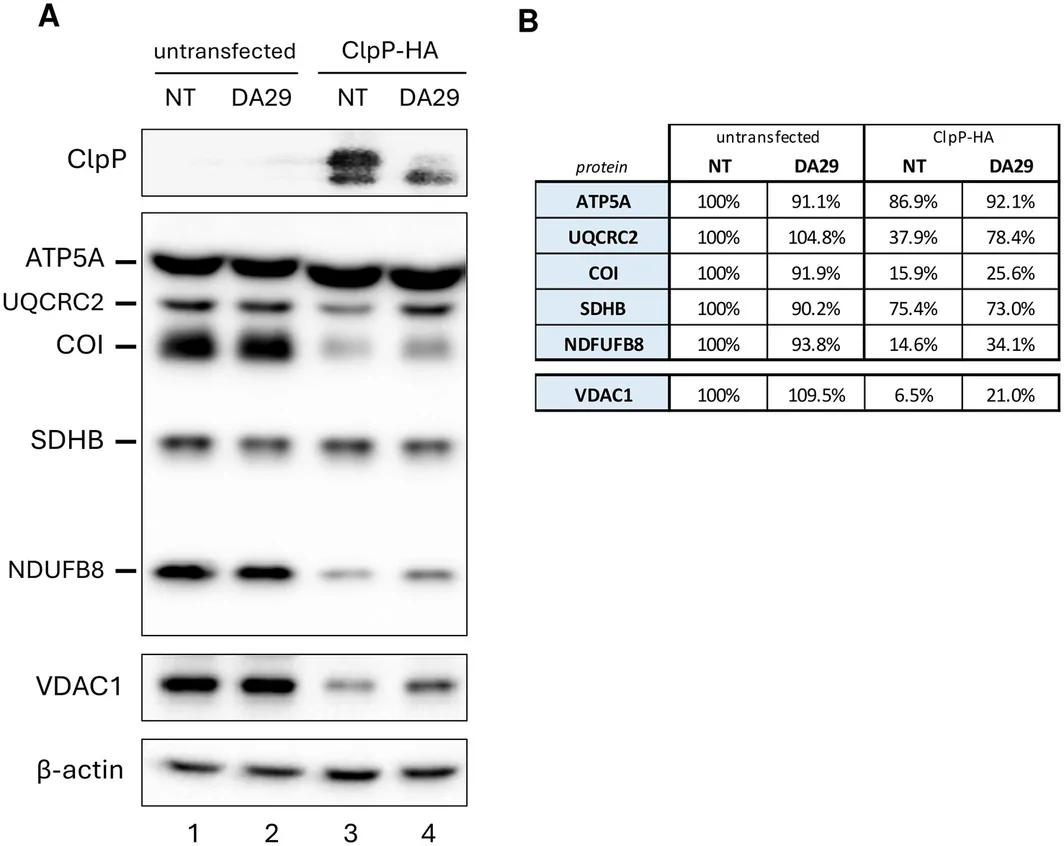
Combined effect of ClpP‐HA expression and DA29 administration in ClpP‐KO cells. (A) Whole‐cell lysates were prepared from untransfected and ClpP‐HA‐overexpressing knockout cells, either untreated (NT) or treated with DA29 (1.5 μM) for 2 days. Aliquots (20 μg) were subjected to immunoblotting, as detailed in Section 2.6. (B) Relative quantification of proteins shown in panel A. For each protein, densitometric analysis was performed on the bands obtained in the different conditions, normalized to β‐actin (loading control) and compared to the untransfected untreated (NT) sample (fixed as 100%); n.d., indicates not detectable signal.

To confirm the above results obtained in ClpP‐HA‐overexpressing cells, we decided to express a GFP‐tagged version of ClpP, exploiting the advantage of in cell fluorescence. The protein overexpressed in wild‐type HEK293T cells had a molecular weight twice that of endogenous ClpP (Figure 5A). Therefore, given the bulkiness of GFP, we first wondered whether the fusion protein was able to assemble, forming the characteristic tetradecameric structure of the active enzyme. To this purpose, protease assembly was qualitatively assessed by isokinetic sucrose gradient analysis (Figure 5B). Upon transient expression, ClpP‐GFP was found distributed across the entire gradient, including the heavier fractions (from 7 to 11) where large protein complexes sediment. Interestingly, endogenous ClpP displayed a co‐migration profile alongside ClpP‐GFP across the gradient. This overlapping sedimentation profile qualitatively suggests that both endogenous ClpP and ClpP‐GFP might reside within the same large complexes. In the absence of targeted experiments, however, we cannot in principle exclude the presence of partially aggregated or misfolded GFP fusion versions in the denser fractions. Notably, in untransfected cells ClpX and ClpP co‐sedimented, whilst in ClpP‐GFP‐overexpressing cells ClpX accumulated in the top gradient fraction (Figure 5B), which contains low‐molecular‐weight components such as free proteins that are not engaged in the assembly of larger complexes. Therefore, in these cells the chaperone component is probably not involved in the formation of high‐molecular‐weight ClpP complexes.

**FIGURE 5:**
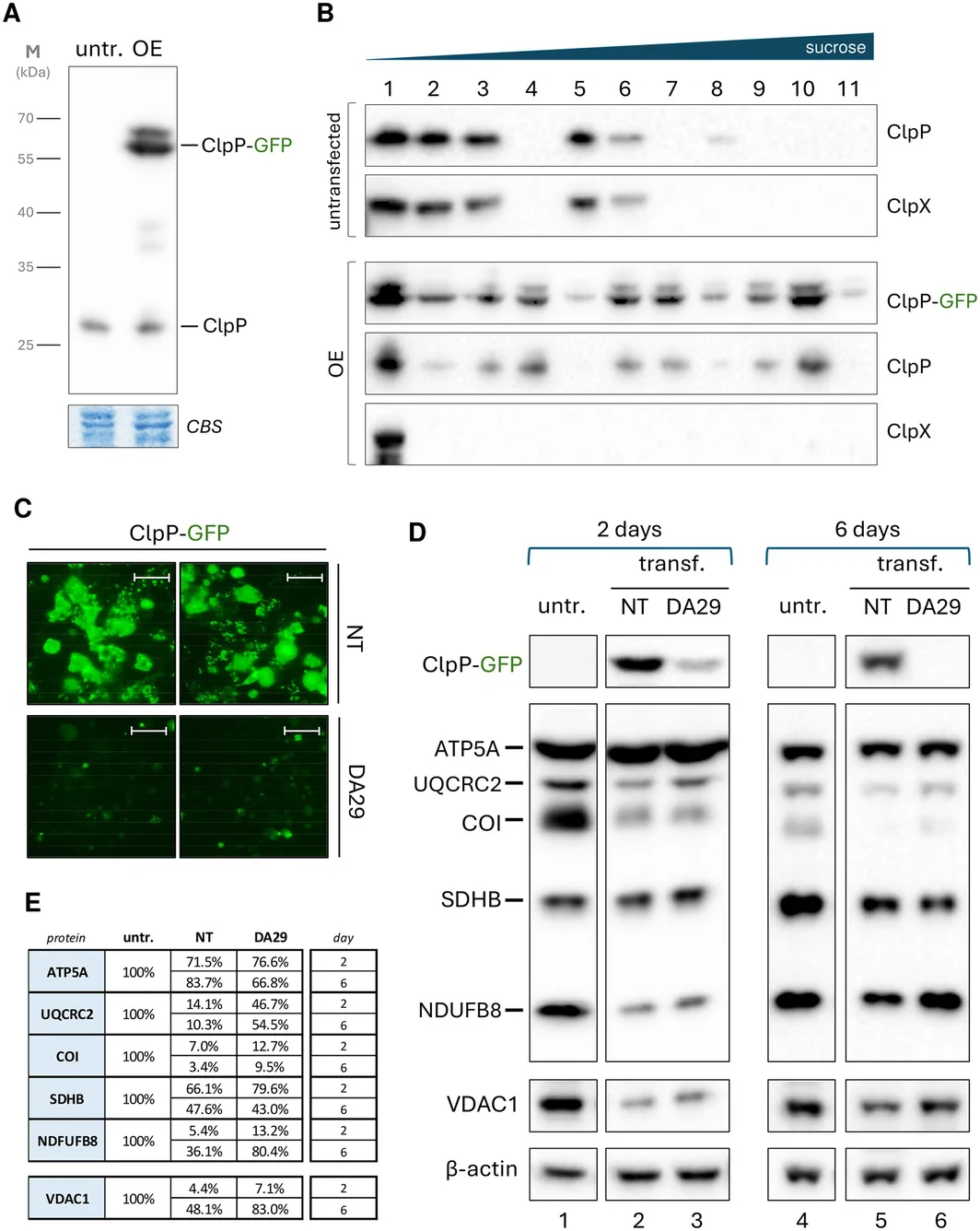
Molecular analysis of ClpXP complex upon GFP‐tagged ClpP expression. (A) Immunoblotting of whole‐cell lysates (30 μg) from untransfected (untr.) and ClpP‐GFP‐overexpressing (OE) wild‐type cells. CBS of total proteins was used as a loading control. Molecular weight markers (M; PageRuler Prestained Protein Ladder, Thermo Fisher Scientific) are indicated to the left. (B) Isokinetic sucrose gradient followed by immunoblot analysis of ClpP (endogenous and GFP‐tagged) and ClpX in either untransfected (upper panels) or ClpP‐GFP‐overexpressing (OE, lower panels) wild‐type cells. Whole‐cell lysate (1 mg) was centrifuged on a 10%–30% gradient using the SW 60Ti rotor at 40000 rpm for 2 h and 15 min at 4°C, and subjected to immunoblotting. (C) Fluorescent signal of ClpP‐GFP expressed in ClpP‐KO cells followed by 3‐day DA29 treatment. For each sample (untreated and DA29), two representative images are shown (white scale bar: 20 μm). (D) Whole‐cell lysates were prepared from untransfected (untr.) and ClpP‐GFP‐overexpressing (transf.) ClpP‐KO cells, either untreated (NT) or treated with DA29 (1.5 μM) for 2 and 6 days. Aliquots (20 μg) were subjected to immunoblotting, as detailed in Section 2.6. The 2‐day untransfected control (lane 1) is the same as shown in Figure 4A (lane 1). Data were obtained from two independent experiments, one for each time point. (E) Relative quantification of proteins shown in panel D. At the two time points, for each protein, densitometric analysis was performed on the bands corresponding to the untreated (NT) and DA29‐treated samples, normalized to β‐actin (loading control) and compared to the untransfected (untr.) sample (fixed as 100%).

The effect of transient ClpP‐GFP overexpression was analyzed in knockout cells, followed by DA29 treatments for 2 and 6 days. Fluorescent imaging showed an almost complete switch‐off of the GFP signal in DA29‐treated cells compared to untreated (Figure 5C), coherently with the decrease in the ClpP‐GFP levels observed by immunoblotting analysis (Figure 5D). The 2‐day ClpP‐GFP overexpression caused a general decline in the respiratory subunits levels (Figure 5D, lane 2 and Figure 5E) compared to untransfected (lane 1). DA29 treatment partially restored the observed decreased protein levels, except for SDHB (lane 3 vs. lane 2), mirroring the ClpP‐HA overexpression data (see Figure 4); an even more evident recovery was observed after 6 days (Figure 5D, lane 6 vs. lane 5 and Figure 5E).

Overall, the reported data indicate that ClpP‐GFP was proteolytically active and confirm that prolonged hyperactivation of ClpP in non‐tumor cells induced its degradation and the recovery of OXPHOS subunits, pointing to a cellular mechanism of protection from uncontrolled proteolysis. The implications of these findings will be explored in detail in the Discussion section.

## 4 Discussion

Diffuse intrinsic pontine glioma (DIPG) is an aggressive cancer that primarily affects children, developing in the pons region of the brainstem. Although research has been improving our understanding of the disease, no targeted treatments have yet produced a meaningful increase in patient survival. Traditional chemotherapy has had minimal impact, and while radiotherapy remains the standard treatment, it offers only temporary symptom relief and does not provide a cure [15]. From a molecular perspective, the most promising pharmacological approach involves the hyperactivation of ClpP [16], a mitochondrial protease responsible for protein quality control inside the mitochondrial matrix of different multicellular eukaryotes [26, 27, 28, 35, 36]. Several drugs, including the FDA‐approved ONC201, have been demonstrated to enhance ClpP proteolytic activity, resulting in extensive degradation of key mitochondrial proteins and disruption of the respiratory chain [18, 19, 28, 37, 38]. Prolonged mitochondrial stress induced by ClpP hyperactivation seems to selectively push tumor cells toward apoptosis, while normal cells appear more resilient, possibly because they do not strictly rely on OXPHOS to meet their energy demands. However, resistance to ONC201 has been observed in some diffuse glioma patients, which raises important questions about the metabolic adaptations that might allow tumor cells to escape ClpP‐targeted therapies. Therefore, finding new compounds that are easier to synthesize, less likely to run into resistance issues, and still capable of precisely targeting ClpP is crucial. We and our collaborators have recently identified DA29, a small piperazine‐based compound that is able to hyperactivate ClpP. In both patient‐derived cell lines and organoids, DA29 shows activity comparable to ONC201 and does not bind D2/D3 receptors [20], which is a meaningful pharmacological distinction [39].

To further investigate the mechanism by which DA29 activates ClpP in mitochondria and considering the limitations due to patient‐derived DIPG cells, we carried out the analyses in HEK293T cells, a well‐established non‐tumor cellular model widely used in pharmacological studies as a control. Unlike cancer cells, HEK293T are metabolically distinct and do not strictly depend on ClpP‐mediated mitochondrial quality control for their survival. In DIPG cells, mechanistic analyses have been generally limited to the first 24 h of treatment, as the rapid cytotoxicity induced by DA29 leads to extensive cell death at earlier time points, preventing the reliable investigation of adaptive molecular responses. In contrast, the prolonged viability of HEK293T cells at high doses of ClpP activators [16] allowed us to extend the analysis over time and monitor the dynamic cellular response to protracted ClpP activation, complementing the analyses recently reported by us and our collaborators on the effect of DA29 treatment on patient‐derived DIPG cells and organoids [20].

The ability of DA29 to activate endogenous ClpP was evaluated in the protease mitochondrial environment. Activators are commonly selected during screening based on their ability to enhance the activity of human ClpP recombinant protease [38], which, although correctly assembled, lacks its chaperone ClpX. To test ClpP proteolytic activity in its physiological context, a mitochondrial lysate was used as a source of ClpP activity, demonstrating that DA29 was able to activate the protease. Importantly, DA29 specifically enhanced ClpP activity, as demonstrated using mitochondrial lysates prepared from ClpP‐KO cells. DA29 treatment of cultured cells also confirmed this selectivity: the compound was cytotoxic in wild‐type cells but not in knockout cells, in contrast to DA28 that did not activate the protease in the fluorescent assay with mitochondrial lysates and exhibited no cellular toxicity, suggesting that DA29 specifically targets ClpP.

Administration of DA29 induced the same phenotype observed in patient‐derived DIPG cells, namely a substantial reduction in the amount of mtDNA and subunits representing the different respiratory chain complexes and mitoribosomes. An interesting aspect emerged from the time‐course analysis of DA29 molecular effects. Prolonged DA29 treatment resulted in a fluctuation in ClpP levels, which declined to undetectable after 3 days and then increased slightly after 6 days. It is intriguing that ClpP hyperactivation, in addition to degrading protease substrates and inducing mitochondrial dysfunction, also led to a reduction in protease levels.

What is the mechanism underlying this negative feedback loop? A possible explanation might be ClpP self‐digestion as a consequence of uncontrolled activity due to hyperactivation [40]. Additionally, other mitochondrial proteases might intervene to lower ClpP levels when it becomes overly active, thereby preserving the physiological quality control of mitochondrial proteins. Although purely speculative and so far untested, this regulatory role might be covered by matrix proteases, such as LONP1, which is important for mitochondrial protein regulation [7, 11, 41] and shows a functional crosstalk with ClpP, cooperating to reduce proteotoxic stress and maintain mitochondrial functionality [10, 42, 43]. Overall, it can be speculated that ClpP hyperactivation may trigger a negative regulatory feedback loop that, in turn, could contribute to long‐term drug resistance (Figure 6).

**FIGURE 6:**
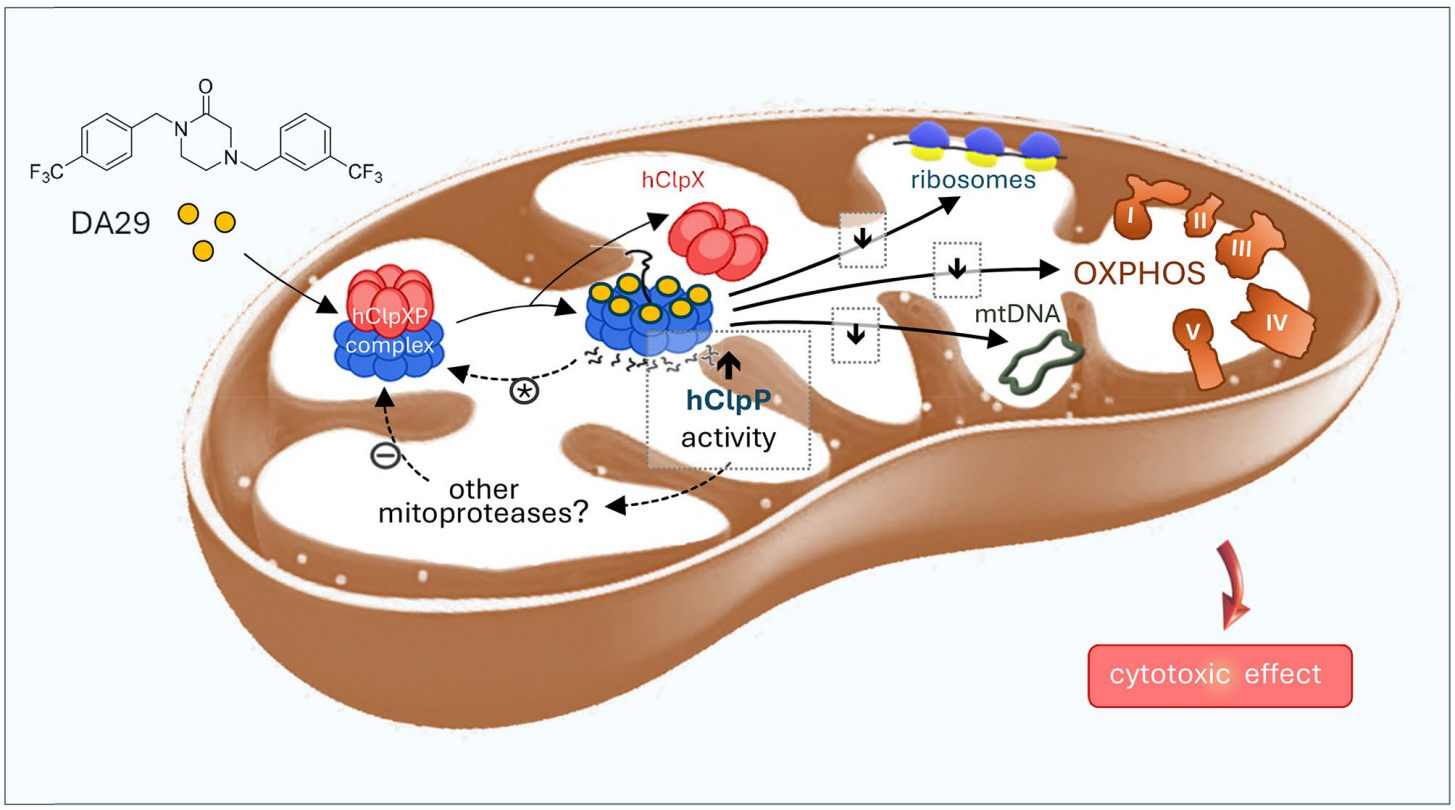
Hypothetical model of the mitochondrial regulatory network involving the protease ClpP chemoactivated by DA29. DA29‐mediated chemoactivation of ClpP affects mtDNA, mitoribosomes, and OXPHOS subunits. Significant fluctuation in ClpP levels in response to DA29 administration might determine opposite cellular effects, such as toxicity or drug resistance. Solid arrows represent the experimental evidence described in this manuscript; dashed arrows represent the hypothesized negative feedback mechanism involving ClpP level fluctuation. Depicted symbols: ‐, degradation; *, self‐degradation; ↑, up‐regulation; ↓, down‐regulation.

Although there is no apparent direct link between ClpP and regulation of mitochondrial biogenesis, it has been demonstrated that in ClpP^−/−^ mice adipocytes the protease deficiency leads to upregulated markers of mitochondrial biogenesis and mass, contributing to a paradoxical beneficial metabolic effect [44]. Recently, research conducted on primary cell lines derived from diffuse midline glioma patients who had developed resistance to imipridone therapy suggests that increased mitochondrial biogenesis and mtDNA levels may constitute an intrinsic resistance mechanism [45]. The observed increased VDAC1 level in ClpP‐overexpressing cells exposed to DA29 (see Figure 4, lane 4, and Figure 5D, lanes 3 and 6) aligns with the possibility that enhanced mitochondrial biogenesis contributes to a hypothetical drug‐resistance pathway associated with ClpP degradation.

Further noteworthy observations were obtained by overexpressing exogenous ClpP in knockout cells. We observed that the expression of C‐terminally tagged versions of ClpP mimicked the effect of ClpP activators by depressing the levels of the analyzed respiratory chain subunits, except for SDHB. Hypothetically, SDHB's reluctance to degradation by overexpressed‐tagged ClpP might be due to structural protection as the B subunit is tightly packed into Complex II between SDHA and the transmembrane subunits SDHC/D [46]. To make SDHB more accessible to ClpP proteolytic chamber, the presence of the ClpX unfoldase would be required. The experimental observation that ClpX does not assemble with the overexpressed‐tagged protease (see Figure 5B), for reasons yet to be clarified, could explain SDHB selective preservation. The alteration of ClpXP complex assembly and ClpP/ClpX stoichiometry could represent a limitation of ClpP‐overexpressing models; nevertheless, the overexpressed phenotype mimics the effect mediated by DA29 treatment on wild‐type cells.

Data presented here indicate that elevated ClpP activity in mitochondria, whether due to chemoactivation or transfection‐achieved elevated protease levels, impairs mitochondrial function. Intriguingly, prolonged DA29‐mediated activation of overexpressed ClpP in knockout cells partially rescued the mitochondrial phenotype, with the concomitant depletion of exogenous ClpP itself. Therefore, upon overexpression, we hypothesize a threshold beyond which the proteolytic activity of ClpP could be slowed down through its degradation, with consequent recovery of mitochondrial OXPHOS subunits, particularly NDUFB8, and mitochondrial mass marker VDAC1. Translating these findings to tumor cells presents some limitations: in these cells, ClpP is widely expressed but exhibits substantial heterogeneity and is often associated with features that lack prognostic significance. Consistently, therapeutic outcomes appear to be highly context‐dependent [12]. Nonetheless, similarly to the ClpP‐overexpressing HEK293T cellular model, many tumor cells display high ClpP levels, making hyperactivation lethal within a relatively short timeframe. However, in certain ClpP‐rich tumor cells, which escape immediate cell death, prolonged hyperactivation may lead to a reduction in protease levels, thereby diminishing the efficacy of the activators and ultimately promoting their proliferation.

The effects of DA29 on mitochondrial function reported here are consistent with the disruption of mitochondrial homeostasis resulting from ClpP hyperactivation induced by imipridone anticancer drugs (ONC201, ONC212, ONC206) and their simplified analogues (TR compounds), as well as by other recently identified compounds such as ZK53, CLPP‐1071, and THX6 [47]. Conversely, to the best of our knowledge, fluctuations in ClpP levels following chemoactivation have been much less extensively documented in the literature. Treatment of cultured neuroblastoma cells with ONC201 has been reported to reduce ClpP and ClpX levels. Interestingly, in the same study, prolonged treatment of a neuroblastoma xenograft model with a high dose of ONC201 increased immunoreactive ClpP and ClpX levels [48].

Although limited to DA29, our findings raise the possibility that prolonged ClpP hyperactivation elicits an adaptive response aimed at restoring mitochondrial proteostasis. Further studies with different ClpP agonists are needed to determine whether the recovery of ClpP levels represents a general feature of sustained ClpP activation or a unique property of DA29. Moreover, a thorough understanding of the regulatory network underlying mitochondrial proteostasis remains essential for the potential therapeutic use of DA29 and other ClpP agonists to effectively balance ClpP chemoactivation with selective targeting of cancer cells.

## Author Contributions

P.L.P. and F.B. conceived and designed the research; F.R., M.M. and F.B. performed the experiments and acquired the data; M.G.P., A.S., P.L.P. and F.B. analyzed and interpreted the data; M.G.P. and A.S. provided funding; P.L.P. and F.B. drafted the manuscript and all authors revised and edited the manuscript.

## Funding

This work was supported by Ministero dell'Università e della Ricerca (MUR), CN000000013. Ministero della Salute (Italy Ministry of Health), 2022WYFST2. Banca d'Italia (Bank of Italy), XI 3 10.

## Conflicts of Interest

The authors declare no conflicts of interest.

## Data Availability

Data sharing not applicable to this article as no datasets were generated or analyzed during the current study.
